# Adverse Reactions by Radiopharmaceuticals: Retrospective Analysis of the Portuguese National Pharmacovigilance System

**DOI:** 10.1177/10600280251316542

**Published:** 2025-02-23

**Authors:** Sara Martins, Ângelo Jesus, Ricardo Andrade, Mariana Rocha, Ana Martín-Suarez

**Affiliations:** 1 LAQV/REQUIMTE, Escola Superior de Saúde, Instituto Politécnico do Porto, Porto, Portugal; 2 Departamento de Ciencias Farmacéuticas, Universidad de Salamanca, Salamanca, Spain; 3 OWLPharma Consulting, Coimbra, Portugal; 4 Instituto de Investigación Biomédica de Salamanca, Salamanca, Spain

**Keywords:** radioisotopes, drug safety, adverse drug reactions, nuclear medicine, hospital pharmacy

## Abstract

**Background:**

Radiopharmaceuticals are essential in the field of nuclear medicine, but like any other medicinal product, radiopharmaceuticals can potentially cause adverse reactions in patients.

**Objective:**

To describe the adverse reactions to radiopharmaceuticals reported to the Portuguese National Pharmacovigilance System (SNF).

**Methods:**

We performed a retrospective, observational study by examining individual case safety reports (ICSRs) provided by the SNF related to all radiopharmaceuticals commercially available in Portugal from 2010 to 2023.

**Results:**

The SNF received a total of 84 ICSRs. These reports resulted in a total of 224 adverse drug reactions (ADR), which involved a total of 15 different radiopharmaceuticals. The mean age of patients was 61.9 years old. Twenty-one different system organ classes (SOCs) were identified, with the most prevalent situations being “Gastrointestinal Disorders” (18.3%; n = 41) followed by “General disorders and administration site conditions” (16.5%; n = 37), “Skin and subcutaneous tissue disorders” (11.2%; n = 25) and “Blood and lymphatic system disorders” (10.3%; n = 23). Fifty-seven reports (67.85%) showed at least 1 serious ADR. Most notified radiopharmaceuticals were, respectively, radium—223 (n = 36, 41.4%), lutetium-177 oxotreotide (n = 12, 13.8%) and iodide—131 (n = 9, 10.3%).

**Conclusion and Relevance:**

Although the number of notifications is limited, these findings provide valuable insights into the types and frequencies of adverse reactions associated with radiopharmaceuticals used in Portugal between 2010 and 2023. The data highlight the importance of continued pharmacovigilance efforts to monitor the safety of these specialized medical products and inform clinical decision-making.

## Introduction

A radiopharmaceutical is a specialized medication that incorporates a radioactive element, known as a radionuclide, which serves as a tracer. These compounds come in 2 main forms. In some cases, the radionuclide is chemically bound to a pharmacological agent that acts as a carrier. This carrier helps direct the radioactive component to specific areas in the body. Alternatively, certain radiopharmaceuticals consist solely of a radionuclide in its ionic state. In this form, the radionuclide itself can function as the targeting agent, without the need for an additional carrier molecule.^1-3^

Both types of radiopharmaceuticals are designed to deliver radioactivity to specific sites in the body, enabling various diagnostic and therapeutic applications in nuclear medicine. Radionuclides are identified by the type of radiation which they emit (ionizing radiation in the form of electromagnetic waves (γ-rays or X-rays) or in the form of particles (neutrons, β, or α); by the energy of the radiation and by the half-life time. The effective half-life of a radiopharmaceutical is related to both the physical decay of the radionuclide (ie, the time required for the activity of a radionuclide to decrease by decay to half its initial value), and to the biological elimination of the radiopharmaceutical, which refers to the time required for the amount of radiopharmaceutical in the body to be halved. Ideally, the effective half-life should be short enough to minimize patient exposure, but long enough for the radiopharmaceutical to exert its action.^4^

Radiopharmaceuticals are used in nuclear medicine, where they bind to a target organ or tissue, according to the affinity of the ligand, and the radioactive element enables a diagnostic or therapeutic process depending on the radioactive emission.^1^ Radiopharmaceuticals used for diagnosis require radionuclides that emit either positrons (β+) or γ-rays. Gamma-ray emitters are useful for SPECT (single-photon emission computed tomography) imaging, while PET (positron emission tomography) relies on β+ emission. In turn, radiopharmaceuticals used for therapeutic purposes are those that emit electrons by β-decay and those that decay with the emission of α-particles.^1^ Around 95% of radiopharmaceuticals are used for diagnostic purposes, and normally, this type of radiopharmaceutical has no pharmacological effects on the body as trace amounts of radioactive markers are used.^4^ The choice of radiopharmaceutical depends on factors like the patient’s general condition, renal function, type and stage of the disease, and radionuclide properties.^4,5^

Pharmacovigilance of radiopharmaceuticals is a critical aspect of ensuring the safety and efficacy of these specialized drugs. Radiopharmaceuticals are unique, in that, they contain radioactive isotopes that emit radiation for diagnostic or therapeutic purposes.^6^ They are also used in very small dosages when compared with conventional drugs and the frequency of administrating radiopharmaceuticals in an individual during his lifetime is also very low. Nevertheless, due to their specific nature, radiopharmaceuticals require careful monitoring and surveillance to detect and manage any potential adverse effects or safety concerns.^7^ Nuclear pharmacists, nuclear medicine technologists, and other health professionals, play a crucial role in ensuring the safe handling, administration, and monitoring of these specialized drugs.^8^ Their involvement in pharmacovigilance is essential for maintaining patient safety and optimizing the use of radiopharmaceuticals.

Radiopharmaceuticals have a lower reported incidence of adverse reactions compared with conventional drugs.^9^ This could be attributed to their unique characteristics that set them apart from typical pharmaceuticals. Radiopharmaceuticals generally lack significant pharmacological effects and dose-response relationships (notable exception for radiotherapeutic agents) and are typically administered in small doses for a limited number of times to patients.^10,11^ However, the possibility of an adverse event to radiopharmaceuticals cannot be completely ruled out.^10-13^ The British Nuclear Medicine Society maintains an online database of adverse reactions to radiopharmaceuticals (ARRPs), with reported prevalence rates of only 3.1 and 2.5 per 100 000 administrations in 2013 and 2015, respectively.^14^ These reactions are often minor, such as skin rash, pruritus, and vomiting. In contrast, the national pharmacovigilance database of France documented 304 reports of ARRPs between 1989 and 2013, with 43% classified as serious adverse events resulting in 12 deaths, 15 life-threatening complications, 89 hospitalizations, and 15 other serious conditions.^3^ Instances like the withdrawal of technetium-99m (^99m^Tc) fanolesomab due to severe adverse reactions leading to life-threatening events^11^ or the withdrawal of ¹³¹I-lipiodol for unfavorable benefit to risk ratio,^15^ highlight the importance of monitoring and reporting adverse reactions associated with radiopharmaceutical use. The widespread use of radiopharmaceuticals in modern health care has heightened the significance of adverse event reporting. This practice is crucial as it serves to notify health care professionals about potential issues, enables a comprehensive evaluation of the extent and severity of these problems, and enhances the precision of diagnosing adverse reactions. Furthermore, by facilitating the development of effective treatment strategies for these reactions, diligent reporting contributes significantly to mitigating the negative consequences associated with radiopharmaceutical use. Ultimately, this proactive approach to monitoring and addressing adverse events plays a vital role in safeguarding patient well-being and optimizing the benefits of radiopharmaceutical applications in medical practice.^16^

The distinctive characteristics of radiopharmaceuticals, requires specialized training for health care professionals engaged in their pharmacovigilance. Given the unique properties of these drugs, it is crucial that practitioners develop a specific skill set. This includes a thorough understanding of radiation safety principles, proficiency in interpreting imaging studies, and the ability to identify adverse reactions particular to radiopharmaceuticals. To cultivate these essential competencies, targeted training programs are indispensable. Such specialized education ensures that health care professionals are well-equipped to effectively monitor, assess, and manage the safety aspects of radiopharmaceuticals, thereby enhancing patient care and safety in nuclear medicine and related fields. Pharmacovigilance of radiopharmaceuticals is challenging, but overcoming these challenges requires a collaborative effort from stakeholders involved in nuclear medicine and pharmacy to ensure the safe and effective use of radiopharmaceuticals, while prioritizing patient safety.

In this study, the primary aim was to perform an extensive and in-depth examination of individual case safety reports (ICSRs) pertaining to suspected adverse drug reactions (ADRs) linked to radiopharmaceuticals within the Portuguese health care context.

## Methods

This research employed a retrospective, observational approach, focusing on the examination of spontaneous reports submitted to the Portuguese National Pharmacovigilance System (SNF) concerning various radiopharmaceuticals available in the Portuguese market. The study encompassed a comprehensive analysis of data spanning from 2010 to 2023. The data used here were entirely made available by the Directorate for Risk Management for Medicines of INFARMED- National Authority of Medicines and Health Products, I.P,. and all legal and regulatory requirements were met when requesting and using the data for this research.

The variables under study are as follows:

1. Number of cases notified per year.
2. Identification of the medicine—trade name—associated with the ADR.
3. Identification of the source of notification.
4. Demographic characterization of the population affected by the ADRs:
   - Age.
   - Sex.
5. Characterization of the type of ADR:
   - Degree of seriousness (serious, non-serious).
   - Severity criteria.
   - Type of effect/affected organ (system organ class [SOC]-MedDRA).
   - Causality assessment.

The classification of ADRs was coded by SOC according to the MedDRA dictionary (27.1 version).

## Results

Between January 2010 and December 2023, 84 out of 136,112 reports to the SNF were spontaneous reports of suspected ADRs related to radiopharmaceuticals, representing a frequency of 6.17 × 10−4 compared to other drug classes. These 84 reports described a total of 224 ADRs, which involved 15 different radiopharmaceuticals.

### Characterization of Individual Case Safety Reports

The mean age of patients included in the ICSRs was 61.9 years. The youngest patient was 18, while the oldest was 87 years old. Age-related data were not available in 34.5% (n = 29) of the reports. Most reports (36.9%; n = 31) concerned people who were between the ages of 18 and 64, with people 65 and older coming in second (28.6%; n = 24).

**Figure 1.**
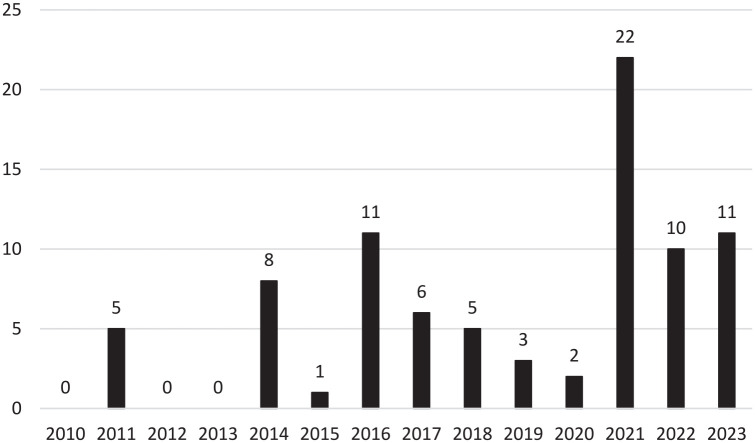
Characterization of the number of individual case spontaneous reports (ICSRs) between 2010 and 2023.

Most of the ICSRs were related to male individuals (62.4%; n = 53), with no information available for this variable in 4.7% (n = 4) of cases. Regarding the reporter, the ICSRs included in this analysis were carried out mostly by physicians, (58.8%; n = 50) (Table 1). The highest number of ICSRs occurred during the year of 2021 (26.2%; n = 22), and no ICSRs were submitted in 2010, 2012, and 2013 (Figure 1).

**Table 1.** Characterization of Patients and Reporters of the Individual Case Safety Reports (ICSRs).

Patient’s age	n	%
18-64 years old	31	36.9%
65 years old or above	24	28.6%
Unknown	29	34.5%
Total	84	100.0%
Patient’s sex	n	%
Male	53	62.4%
Female	27	31.8%
Unknown	4	4.7%
Total	84	100%
Reporter’s professional group	n	%
Physicians	50	58.8%
Pharmacist	15	17.6%
Patient or other non-health care professional	3	3.5%
Other health care professional	16	18.8%
Total	84	100%

Fifteen different radiopharmaceuticals were identified in the ICSRs. Radium (^223^Ra) dichloride was named in 36 (41.4%; n = 36) ICSRs, followed by lutetium (^177^Lu) oxodotreotide (3.8%; n = 12), sodium iodide (^131^I) (10.3%; n = 9) and Technetium (^99m^Tc) oxidronic acid (9.2%; n = 8) (Table 2).

**Table 2.** Characterization of the Suspected Drug Involved the ICSRs.

Radiopharmaceutical	N	%
Radium (^223^Ra) dichloride *	36	41.4%
Lutetium (^177^Lu) oxodotreotide *	12	13.8%
Sodium iodide (^131^I) *	9	10.3%
Technetium (^99m^Tc) oxidronic acid	8	9.2%
Fludeoxyglucose (^18^F)	4	4.6%
Iobenguane (^123^I)	3	3.4%
Gallium (^68^Ga) edotreotide	3	3.4%
Lutetium (^177^Lu) vipivotide tetraxetan *	2	2.3%
Technetium (^99m^Tc) pertechnetate	2	2.3%
Technetium (^99m^Tc) sestamibi	2	2.3%
Ureia (^14^C)	2	2.3%
Lutetium (^177^Lu) DOTATE *	1	1.1%
Chromium (^51^Cr) edetate	1	1.1%
Technetium (^99m^Tc) macrosalb	1	1.1%
Technetium (^99m^Tc) tetrofosmin	1	1.1%
Therapeutic radiopharmaceuticals are marked with a *		

#### Characterization of adverse drug reactions

All the reactions presented in the reports were analyzed for the variables used in the characterization of ADRs, irrespective of whether there might be a relationship solely with the suspected radiopharmaceutical or with other associated drugs in the same report.

For the 84 ICSRs, a total of 224 ADRs were detected. Following the classification of ADR by MedDRA SOC coding, 21 different SOCs were identified, with the most prevalent situations being “Gastrointestinal Disorders” (18.3%; n = 41) followed by “General disorders and administration site conditions” (16.5%; n = 37), “Skin and subcutaneous tissue disorders” (11.2%; n = 25) and “Blood and lymphatic system disorders” (10.3%; n = 23) (Table 3).

**Table 3.** Classification of the ADRs According to MedDRA SOC Classification.

Primary SOC reaction	N	%
Gastrointestinal disorders	41	18.3%
General disorders and administration site conditions	37	16.5%
Skin and subcutaneous tissue disorders	25	11.2%
Blood and lymphatic system disorders	23	10.3%
Investigations	15	6.7%
Nervous system disorders	13	5.8%
Injury. poisoning and procedural complications	12	5.4%
Neoplasms benign, malignant, and unspecified (incl. cysts and polyps)	12	5.4%
Infections and infestations	6	2.7%
Musculoskeletal and connective tissue disorders	6	2.7%
Renal and urinary disorders	5	2.2%
Hepatobiliary disorders	5	2.2%
Eye disorders	4	1.8%
Endocrine disorders	4	1.8%
Psychiatric disorders	4	1.8%
Immune system disorders	3	1.3%
Metabolism and nutrition disorders	3	1.3%
Cardiac disorders	2	0.9%
Vascular disorders	2	0.9%
Respiratory, thoracic and mediastinal disorders	1	0.4%
Surgical and medical procedures	1	0.4%
Total	224	100%

Seriousness criteria were examined for ADRs labeled as “serious.” Fifty-seven reports (67.85%) showed at least 1 serious ADR. A total of 132 ADRs were identified, with 66.4% (n = 81) classified as “clinically important” and 4.9% (n = 6) categorized as “fatal” (Table 4).

**Table 4.** Classification of Suspected ADRs According to Seriousness Criteria.

Seriousness criteria	N	%
Clinically important	81	66.4
Life-threatening	15	12.3
Disability	16	13.1
Hospitalization	14	11.5
Death	6	4.9
Total	132	100

Regarding the evolution of the ADRs (only 217 ADRs had this information available), 37.3% (n = 81) evolved into a cure and 8% (n = 6) resulted in death (Table 5). It should be highlighted that a single report may encompass multiple, distinct ADRs, which can potentially lead to the same outcome within that report. This complexity in reporting underscores the need for careful interpretation of the data. A particularly noteworthy finding is the high proportion of ADRs with unknown outcomes, accounting for 46.5% (62 cases) of the reported reactions.

**Table 5.** Evolution of Adverse Drug Reactions (ADRs).

Evolution	N	%
Unknown	101	46.5
Cured	81	37.3
Persists without recovery	15	6.9
Recovering	12	5.5
Death	6	2.8
Cured with sequelae	2	0.9

Causality assessment, established by the regulatory authority (Table 6), was conducted for 29.5% (n = 66) of the total identified ADRs. Of these, 45.5% (n = 30) were classified as “probable,” equal value was obtained as “possible,” and only 1.5% (n = 1) as “certain.”

**Table 6.** Causality Assessment by the Regulatory Authority.

Classification	N	%
Probable	30	45.5
Possible	30	45.5
Conditional	3	4.5
Certain	1	1.5
Unrelated	1	1.5
Unlikely	1	1.5

Important medical events (IMEs) and designated medical events (DMEs) were also available to assessment. Table 7 depicts the IME, DME, radiopharmaceutical involved, and the causality assessment made by the regulatory authority.

**Table 7.** IME and DME Identified in the ICSR’s.

Radiopharmaceutical	Reaction PT (IME/DME)	Causality assessment
Chromium edetate (^51^Cr)	Renal tubular injury	Not assessed
Sodium iodide (^131^I)	Exophthalmos Hypothyroidism Sialoadenitis Thyrotoxic crisis	Exophthalmos—possible Hypothyroidism—not assessed Sialoadenitis—probable Thyrotoxic crisis—possible
Fluorodeoxyglucose (^18^F)	Bell’s palsy Death Hepatitis toxic Hyperbilirubinemia	Bell’s palsy—unlikely Death—possible Hepatitis toxic—possible Hepatitis toxic—possible
Technetium (^99m^Tc) macrosalb	Type I hypersensitivity	Type I hypersensitivity—possible
Technetium (^99m^Tc) oxidronic acid	Death Hepatitis toxic Hyperbilirubinemia	Death—possible Hepatitis toxic—possible Hyperbilirubinemia—possible
Lutetium (^177^Lu) vipivotide tetraxetane	Thrombocytopenia	Thrombocytopenia—probable
Radium dichloride (^223^Ra)	Neutropenia Thrombocytopenia Death, diverticulitis, malignant neoplasm progression, metastases to adrenals, metastases to bone, metastases to lung, osteomyelitis, osteonecrosis, osteonecrosis of jaw, pancytopenia (DME), prostate cancer metastatic, sepsis	Neutropenia—probable Thrombocytopenia—probable Not assessed
Lutetium (^177^Lu) oxodotreotide	Encephalitis, hepatic artery stenosis, herpes zoster oticus, intestinal metastasis, intestinal obstruction, malignant neoplasm progression, metastases to liver, metastases to pancreas, myelodysplastic syndrome, pancreatic neuroendocrine tumor, plasma cell myeloma, pulmonary tuberculosis, second primary malignancy, small intestine neuroendocrine tumor, thrombocytopenia	Not assessed

#### Deaths after administration of radiopharmaceuticals

During the period 2010 to 2023, 6 deaths were reported after radiopharmaceutical use in Portugal. Of these, 2 occurred with radium (^223^Ra) dichloride, 2 with lutetium (^177^Lu) oxodotreotide, 1 with fluorodeoxyglucose (^18^F), and 1 where radium (^223^Ra) dichloride and fluorodeoxyglucose (^18^F) were used. In none of these cases, there was any direct causation determined.

### Estimation of the Annual Incidence of Reported Adverse Reactions to Radiopharmaceuticals

In accordance with the public records made available by the Portuguese government on *Portal da Transparência*,^17^ there were 307.725 nuclear medicine exams performed both in public hospitals and other associated health care institutions between 2015 and 2023. In accordance with the data in Table 8, the estimated annual incidence varies between 3.6 × 10^−5^and 5.9 × 10^−4^.

**Table 8.** Estimation of the Annual Incidence of Reported Adverse Reactions to Radiopharmaceuticals.

Year	Number of nuclear medicine exams performed	Number of ADR reports of radiopharmaceuticals	Estimated annual incidence
2015	28 159	1	3.6 × 10^−5^
2016	29 503	11	3.7 × 10^−4^
2017	28 913	6	2.1 × 10^−4^
2018	30 689	5	1.6 × 10^−4^
2019	35 938	3	8.3 × 10^−5^
2020	24 871	2	8.0 × 10^−5^
2021	37 122	22	5.9 × 10^−4^
2022	46 141	10	2.2 × 10^−4^
2023	46 389	11	2.4 × 10^−4^

## Discussion

This study analyzed 84 ICSRs of ADRs related to the use of radiopharmaceuticals, reported between 2010 and 2023. These reports encompassed 15 different radiopharmaceuticals and identified a total of 224 ADRs. Notably, a significant decrease in reporting was observed during 2019 and 2020, which may be attributed to the impact of the Covid-19 pandemic. It likely led to a reduction in scheduled medical examinations, consequently affecting the number of radiopharmaceutical administrations and, by extension, the reporting of related adverse events. Similarly, the sudden increase in 2021 may be related to the heightened sensibility of health care professionals for adverse reaction reporting, following the pandemic. This pattern is verifiable in the SNF Dashboard of public pharmacovigilance data, for all ICSRs submitted during this time period.^18^ Most of the ICSRs were submitted by health care professionals. These results are not surprising, since ADR reporting has always been attributed to health care professionals and they are the ones who report the most. With the inclusion of the general population as potential notifiers since 2012 in Europe, we would expect to see a higher total number of notifications, since the aim of including them was to promote more proactive pharmacovigilance systems.^19^ It may be necessary to take more steps to increase public awareness of pharmacovigilance and their involvement in ADR reporting. Most of the reported adverse reactions were included in the Summary of Product Characteristics for the radiopharmaceutical. The major SOC described where “General disorders and administration site conditions,” “Skin and subcutaneous tissue disorders,” “Blood and lymphatic system disorders,” “Gastrointestinal disorders,” which is in consonance with previous findings.^3,12,14,16,20-22^

In our study, the most notified radiopharmaceutical was the therapeutic, alpha-emitter radium-223. Hematologic toxicity and disease progression were the most clinically important ADRs reported. Previous research has focused on real-life data from radium-223 therapy^23^ confirmed that older patients were at increased risk when compared with younger patients. Lutetium (^177^Lu) oxodotreotide was also among the radiopharmaceuticals with the most ADRs reported. It presented a wide range of ADRs from hematological to gastrointestinal and hepatic toxicity. Previous research^24^ has linked lutetium (^177^Lu) oxodotreotide toxicity to peritoneal metastases, pancreatic metastases or pulmonary metastases, and high tumor grade. However, we cannot make such correlation in this case, since no specific information about the patient health status was retrieved from the ICSR. Sodium iodide-131 was the third radiopharmaceutical with most notified adverse reactions, namely, “endocrine disorders” and “gastrointestinal disorders,” like thyrotoxic crisis, radiation thyroiditis, sialadenitis, and dysgeusia. One of the reasons why sodium iodide-131 is so widely reported is probably that it is the most widely administered therapeutic radiopharmaceutical, for both resistant hyperthyroidism and thyroid cancer. It is hypothesized that the majority of ADRs to sodium iodide-131 are caused by the beta radiation of the isotope at high doses. Furthermore, this radiopharmaceutical tends to build up in the salivary glands and can then be released into the pharynx and mouth, where it can be swallowed and enter the esophagus.^25^ We should also point out that this formulation has a hard gelatine capsule contains some excipients (sodium thiosulfate pentahydrate, disodium phosphate dihydrate, and sodium hydroxide) that can cause some adverse reactions.^9^ Technetium (^99m^Tc) oxidronic acid was the fourth radiopharmaceutical on our list with the most ADRs reported. One reason for this may be because they have been the most widely used radiopharmaceuticals in SPECT imaging for many years.^3,9^ The most frequent ADRs associated with this drug are erythematous maculopapular rashes, nausea, pruritus, hypotension, fever, and headaches.

The limited data of adverse events make it challenging to detect rare or unexpected reactions, as large data sets are often needed to identify signals for uncommon adverse events.^11-13,26^ Moreover, the substantial gap in follow-up information highlights a significant challenge in comprehensive ADR monitoring and emphasizes the importance of improving post-reaction tracking and reporting mechanisms, to enhance our understanding of radiopharmaceutical safety profiles. These drugs are normally regarded as safe medicines. A French study^3^ reports that ARRPs were 6.2 × 10^−4^ of that with other classes of drugs. This finding closely aligns with the results obtained in our study, suggesting a consistent pattern in the safety profile of radiopharmaceuticals across different populations.

Various international studies and regulatory bodies have reported consistent findings regarding the frequency of ARRPs. The European Association of Nuclear Medicine, the Japanese Authorities, and several US researchers’ groups provide a similar frequency of ARRPs reported, ranging from 0 to 11 × 10^−5^ radiopharmaceutical administrations.^22,27,27-30^ A meta-analysis conducted in 2012, of studies on ARRPs occurrence, determined a global incidence of 1.9 × 10^−5^ administrations.^31^ In our analysis, in Portugal, the annual incidence of reported adverse reactions ranged from 3.6 × 10^−5^and 5.9 × 10^−4^ administrations. This value, although slightly higher, may be attributed to the growing use of radiopharmaceuticals in recent years. As their application becomes more widespread, there is naturally a greater opportunity for adverse reactions to be observed and reported.

The reported ADRs data must be interpreted with caution, acknowledging the potential for underreporting. Not all adverse events are systematically documented or communicated through official channels, which means the actual incidence of ADRs could be higher than the recorded figures. This underreporting phenomenon is a well-recognized limitation in pharmacovigilance, stemming from various factors. Health care professionals may face obstacles in reporting, due to lack of time to complete reporting forms, lack of awareness about the reaction, improper reporting system, overall poor reporting culture, anxiety about potential liability, beliefs that there will be little interest in an already known adverse reaction, and misrecognition or lack of recognition of adverse reactions due to delayed appearance.^3,32-34^ This underreporting can hinder the comprehensive understanding of the safety profile of radiopharmaceuticals. Determining the causal relationship between an adverse reaction and the administration of a radiopharmaceutical is also a challenge. Various factors need to be considered, including the patient’s medical history, concomitant medications, and the specific characteristics of the radiopharmaceutical itself. Conducting thorough causality assessments requires specialized knowledge and expertise in nuclear medicine pharmacovigilance, and of course depends on the information provided in the pharmacovigilance report.^26^ We would like to point out that the causality assessment, which is not a mandatory field in the notification/processing of ADR notifications, is only carried out by the Portuguese National Competent Authority for notifications that it receives directly from citizens and health professionals and that are considered serious. All notifications that are sent directly to EudraVigilance (and forwarded to the respective national pharmacovigilance agencies) by the holder of the marketing authorization (MA), will only have the causality field filled in, if the MA holder has taken this action and coded the respective field.

This study is not without constraints. We are working with a limited amount of data, regarding drugs that can be used both in diagnostic and treatment options, used in very small dosages, with significant differences in the type and energy of the radiation emitted, and mostly used for patients with severe illness. No information about patient’s further medication is analyzed and the assessment for causality is also limited. It might also be legitimate to wonder what proportion of known and expected adverse reactions (especially to therapeutic radiopharmaceuticals) go unreported. This might represent a large proportion of ADRs, which would distort the vision of reality provided by these data. The significance of this research area persists and is growing in importance, paralleling the expanding use of radiopharmaceuticals in medical practice. This trend is particularly evident in the field of targeted radionuclide therapies, with ^177^Lu-vipivotide tetraxetan serving as a prime example. Despite its infrequent use during the early stages of the study, this specific radiopharmaceutical has since seen a dramatic surge in clinical practice, highlighting the rapidly evolving landscape of nuclear medicine and the need for ongoing research. Moreover, we acknowledge that our study was conducted in a country with a population of approximately 10 million. While this provides valuable local insights, the relatively small population size compared with larger regions, such as the United States or the European Union (EU) as a whole, may limit the generalizability of findings.

Further steps would include an analysis of signals generated by radiopharmaceuticals, discussed at Pharmacovigilance Risk Assessment Committee (PRAC). Each month, the PRAC analyzes, prioritizes, and evaluates safety signals concerning medicinal products authorized in the EU. This assessment may result in various recommendations, including an update of the product information. In addition, it would be interesting to review the Electronic Reaction Monitoring Report to review any signal of disproportionate reporting (SDR). SDRs could potentially indicate the presence of a new ADR for the product or a known ADR reported more than expected in the database.^35^

## Conclusion and Relevance

This study focused on a comprehensive analysis of ICSRs of suspected ADRs associated with radiopharmaceuticals, in Portugal. By focusing on ICSRs, the research aimed to capture detailed information about specific incidents, including the nature and severity of reactions, patient demographics, and the circumstances surrounding each event. This approach enabled us to capture a comprehensive understanding of the adverse events associated with radiopharmaceuticals in the Portuguese context. This localized focus, can offer valuable insights for Portuguese health care providers and regulators while also contributing to the broader global understanding of radiopharmaceutical safety. A total of 84 ICSRs describing 224 ADRs involving 15 different radiopharmaceuticals were retrieved. The most prevalent ADRs were related to gastrointestinal disorders, general disorders and administration site conditions, skin and subcutaneous tissue disorders, and blood and lymphatic system disorders. Notably, 57 reports (67.85%) included at least 1 serious ADR. Nevertheless, both frequency and estimated annual incidence of the ADRs were low, and in line with previous research. While data are limited, this comprehensive analysis serves multiple purposes: it can inform clinical practice, guide policy decisions, enhance pharmacovigilance efforts, and ultimately contribute to improving patient safety in nuclear medicine procedures. Moving forward, the results of this study can serve as a foundation for further research. Ongoing monitoring, reporting, and analysis of adverse reactions are crucial to enhance patient safety and ensure the continued benefits of the use of radiopharmaceuticals.

## References

[1] Theobald T . Sampson’s Textbook of Radiopharmacy. 4th ed. Pharmaceutical Press; 2011.

[bibr2-10600280251316542] Coura-Filho GB Torres Silva de Oliveira M Morais de Campos AL . Basic principles of radiopharmaceuticals. Nuclear Medicine in Endocrine Disorders. Springer; 2022:3-7. doi:10.1007/978-3-031-13224-7_1

[3] Laroche ML Quelven I Mazère J Merle L. Adverse reactions to radiopharmaceuticals in France: analysis of the national pharmacovigilance database. Ann Pharmacother. 2014;49(1):39-47. doi:10.1177/1060028014558153 25366341

[4] Saha GB . Fundamentals of Nuclear Pharmacy. 6th ed. Springer; 2010. Accessed January 31, 2025. http://www.amazon.com/Fundamentals-Nuclear-Pharmacy-Saha/dp/1441958592

[5] Lange R Schreuder N Hendrikse H . Radiopharmaceuticals. Practical Pharmaceutics: An International Guideline for the Preparation, Care and Use of Medicinal Products, Second Edition. Springer; 2023:531-550. doi:10.1007/978-3-031-20298-8_23

[6] Meher BR Agrawal K Padhy BM. The global perspective of pharmacovigilance in nuclear medicine practice. Indian J Nucl Med. 2018;33(4):269-272. doi:10.4103/IJNM.IJNM_103_18 30386045 PMC6194762

[7] Dhoundiyal S Srivastava S Kumar S , et al Radiopharmaceuticals: navigating the frontier of precision medicine and therapeutic innovation. Eur J Med Res. 2024;29(1):26. doi:10.1186/S40001-023-01627-0 38183131 PMC10768149

[8] Laven DL. Nuclear pharmacy: potential roles for the technician. J Pharm Technol. 1987;3(1):24-33. doi:10.1177/875512258700300110

[9] Pérez-Iruela JA Pastor-Fructuoso P De Gracia-Rodríguez C Soler-Vigil M Del Val Gómez-Martínez M. Adverse reactions to radiopharmaceuticals. Farmacia Hospitalaria. 2021;45(3):142-149. doi:10.7399/FH.11669 33941058

[10] Hesslewood SR Keeling DH. Frequency of adverse reactions to radiopharmaceuticals in Europe. Eur J Nucl Med. 1997;24(9):1179-1182. doi:10.1007/BF01254254 9283115

[bibr11-10600280251316542] Meher BR Agrawal K Gnanasegaran G. Review of adverse reactions associated with the use of common diagnostic radiopharmaceuticals. Indian J Nucl Med. 2021;36(2):163-167. doi:10.4103/IJNM.IJNM_219_20 34385787 PMC8320829

[bibr12-10600280251316542] Schreuder N Koopman D Jager PL Kosterink JGW van Puijenbroek E. Adverse events of diagnostic radiopharmaceuticals: a systematic review. Semin Nucl Med. 2019;49(5):382-410. doi:10.1053/j.semnuclmed.2019.06.006 31470933

[13] Schreuder N Jacobs NA Jager PL Kosterink JGW van Puijenbroek EP. Patient-reported adverse events of radiopharmaceuticals: a prospective study of 1002 patients. Drug Saf. 2021;44(2):211-222. doi:10.1007/S40264-020-01006-2/TABLES/6 33094442 PMC7847431

[14] Kennedy-Dixon TG Gossell-Williams M Cooper M Trabelsi M Vinjamuri S. Evaluation of radiopharmaceutical adverse reaction reports to the British Nuclear Medicine Society from 2007 to 2016. J Nucl Med. 2017;58(12):2010-2012. doi:10.2967/jnumed.117.194092 28522742

[15] Jouneau S Vauléon E Caulet-Maugendre S , et al 131I-Labeled lipiodol-induced interstitial pneumonia: a series of 15 cases. Chest. 2011;139(6):1463-1469. doi:10.1378/CHEST.10-1591 20947651

[16] Battal H Ozer AY. Adverse reactions to radiopharmaceuticals: liver radiopharmaceuticals. Nucl Med Commun. 2021;42(4):352-359. doi:10.1097/MNM.0000000000001355 33346605

[17] ACSS. Exames realizados por área MCDT. Portal Da Transparência. Published May 21, 2024. Accessed May 21, 2024. https://transparencia.sns.gov.pt/explore/?sort=modified&refine.keyword=MCDT

[18] INFARMED. Desempenho do SNF—INFARMED, I.P. Published 2024. Accessed June 9, 2024. https://www.infarmed.pt/web/infarmed/entidades/medicamentos-uso-humano/farmacovigilancia/desempenho-do-snf

[19] Inácio P Cavaco A Airaksinen M. The value of patient reporting to the pharmacovigilance system: a systematic review. Br J Clin Pharmacol. 2017;83(2):227-246. doi:10.1111/BCP.13098 27558545 PMC5237689

[20] Matos C Härmark L van Hunsel F. Patient reporting of adverse drug reactions: an international survey of national competent authorities’ views and needs. Drug Saf. 2016;39(11):1105-1116. doi:10.1007/s40264-016-0453-6 27581398

[bibr21-10600280251316542] Silberstein EB. Prevalence of adverse events to radiopharmaceuticals from 2007 to 2011. J Nucl Med. 2014;55(8):1308-1310. doi:10.2967/jnumed.114.138057 24876206

[22] Hesslewood S. European system for reporting adverse reactions to and defects in radiopharmaceuticals: annual report 2001. Eur J Nucl Med Mol Imaging. 2003;30:BP87-BP98. doi:10.1007/s00259-003-1342-7 11976816

[23] Tema G Lombardo R Voglino O , et al Adverse events related to radium-223 treatment: “real-life” data from the Eudra-Vigilance database. Minerva Urol Nephrol. 2021;73(3):342-348. doi:10.23736/S2724-6051.20.03690-5 32083419

[24] Dubois J Tosato G Garrigue P Taieb D Guillet B Nail V. Short-term biological toxicity prediction of [177lu] lutetium-oxodotreotide: an original retrospective analysis. Cancer Biother Radiopharm. 2024;39(5):381-389. doi:10.1089/CBR.2023.0195 38655905 PMC11304756

[25] Jané Soler P Pérez Iruela J Gómez Martínez M Lorente Castro B. Mucositis orofaríngea y esofágica: una complicación infrecuente postratamiento ablativo con 131 I—Introducción—Alasbimn Journal. Alasbimn Journal. Published 2018.

[26] Lucas S Ailani J Smith TR Abdrabboh A Xue F Navetta MS. Pharmacovigilance: reporting requirements throughout a product’s lifecycle. Ther Adv Drug Saf. 2022;13:1-16. doi:10.1177/20420986221125006 PMC9520146 36187302

[27] Silberstein EB. Prevalence of adverse reactions to positron emitting radiopharmaceuticals in nuclear medicine. J Nucl Med. 1998;39(12):2190-2192. Accessed May 21, 2024. https://jnm.snmjournals.org/content/39/12/2190 9867168

[bibr28-10600280251316542] Matsuda H Uehara T Okazawa H , et al Full report on a survey of adverse reactions to radiopharmaceuticals from 1975 to 2017 in Japan. Ann Nucl Med. 2020;34(4):299-304. doi:10.1007/S12149-020-01439-W 31989466

[bibr29-10600280251316542] Keeling DH Sampson CB. Adverse reactions to radiopharmaceuticals. United Kingdom 1977-1983. Br J Radiol. 1984;57(684):1091-1096. doi:10.1259/0007-1285-57-684-1091 6509288

[30] EANM Committee on Radiopharmaceuticals. European system for reporting adverse reactions to and defects in radiopharmaceuticals: annual report 1994. Eur J Nucl Med. 1995;22(11):BP29-BP33. doi:10.1007/BF00801632

[31] Salvatori M Treglia G Mores N. Further considerations on adverse reactions to radiopharmaceuticals. Eur J Nucl Med Mol Imaging. 2012;39(8):1360-1362. doi:10.1007/S00259-012-2120-1 22526962

[32] Al Meslamani AZ . Underreporting of adverse drug events: a look into the extent, causes, and potential solutions. Expert Opin Drug Saf. 2023;22(5):351-354. doi:10.1080/14740338.2023.2224558 37300402

[bibr33-10600280251316542] García-Abeijon P Costa C Taracido M Herdeiro MT Torre C Figueiras A. Factors associated with underreporting of adverse drug reactions by health care professionals: a systematic review update. Drug Saf. 2023;46(7):625-636. doi:10.1007/S40264-023-01302-7/FIGURES/2 37277678 PMC10279571

[34] Silberstein EB Ryan J ; Pharmacopeia Committee of the Society of Nuclear Medicine. Prevalence of adverse reactions in nuclear medicine. J Nucl Med. 1996;37(1):185-192. 8543992

[35] Sardella M Lungu C. Evaluation of quantitative signal detection in EudraVigilance for orphan drugs: possible risk of false negatives. Ther Adv Drug Saf. 2019;10:1-11. doi:10.1177/2042098619882819 PMC6804351 31673326

